# Can animal data predict human outcome? Problems and pitfalls of translational animal research

**DOI:** 10.1007/s00259-012-2175-z

**Published:** 2012-07-13

**Authors:** Marianne I. Martić-Kehl, Roger Schibli, P. August Schubiger

**Affiliations:** 1 Collegium Helveticum ETH and UZH, Schmelzbergstrasse 25, 8092 Zurich, Switzerland; 2 Center for Radiopharmaceutical Sciences ETH, PSI and USZ, Zurich, Switzerland

## Introduction

Animal models are believed to be predictive for drug development in human health care. While LD_50_ studies of drug candidates have always been performed in animals like rodents or dogs, efficacy and adverse effect studies using animals as a model for humans were prompted only in the late 1950s, as a consequence of the thalidomide scandal, where thousands of babies were born with severe extremity deformations.

In the last 10–15 years, preclinical research methodologies have increased crucially regarding measurement of sensitivity and specificity. Adapted and improved for dedicated use in animal research, positron emission tomography (PET) and single photon emission computed tomography (SPECT) are good examples of such methodologies. It seems to us that increased measurement precision in animal research has somewhat raised expectations regarding human outcome prediction of preclinical data.

In recent years, on the other hand, there has been increasing scepticism about the essentiality of animal models for medical progress [1–7]. Statements like “virtually every achievement of the last century has depended directly or indirectly on the research with animals” are often found in the literature to emphasize the importance and necessity of animal models used in drug development and medical science. Robert Matthews [6] discussed the validity of this particular statement in a critical article in 2008 and concluded that this statement is anecdotal and does not generally hold true. He is convinced, though, and surely there is evidence that “animal models can and have provided many crucial insights that have led to major advances in medicine and surgery”. Hence, he claims that systematic investigations on the use of animal models and on the evidence that they possibly can provide are necessary.

Gill Langley, in her critical paper in 2009, referred to the same statement as Matthews a year earlier [7]. Langley concluded that relying on animal surrogates of human illnesses is a flawed approach in science. Her own investigations, as well as several published systematic reviews of the reliability of animal models, have shown that fewer than 50 % of animal studies have predicted human outcomes sufficiently. In certain fields of research, e.g. vaccination development against acquired immunodeficiency syndrome (AIDS), prediction failure of chimpanzee and macaque models is 100 % [7, 8].

We are convinced that animal models can be useful tools in biomedical research, but undoubtedly, it has frequently been observed that effects found in animal models cannot be translated to the clinic.

## Translation problems

Sena and colleagues reported only three interventions for acute ischaemic stroke treatment with a convincing effect in patients out of 494 interventions that showed a positive effect in animal models [9]. They have tried to identify reasons for this tremendous discrepancy between preclinical animal studies and patient studies in the clinic. The first category of reasons for the discrepancy is specific for experimental stroke studies, such as schedule of treatment, the co-administration of neuroprotective anaesthetics or the animal models themselves. Animals used in experimental stroke research are mostly young and healthy, whereas patients are often elderly with comorbidities like diabetes and hypertension. Furthermore, in animal studies, time to treatment was often as short as 10 min. Patients for clinical trials, on the other hand, were treated up to 24 h after stroke onset [9].

For many diseases, underlying mechanisms are unknown, which makes it rather difficult to develop representative animal models. Animal models, therefore, are often designed according to observed disease symptoms or show disease phenotypes differing crucially from human ones if underlying mechanisms are being reproduced genetically [7]. A prominent example is the genetic modification of mice to develop human cystic fibrosis in the early 1990s. Unexpectedly, mice showed different symptoms from human patients [10]. A more recent example of non-predictive animal models was described by Ebos et al. in the field of angiogenesis inhibiting agent development against different types of cancer [11]. The authors describe how short-term application of anti-angiogenesis drugs, such as sunitinib, leads to increased metastasis in animal models and consequently to a reduced median survival compared to vehicle-treated groups. In contrast to these findings stand encouraging results after applying sustained sunitinib treatment to animal models with preestablished tumours. This initial research, though, has not considered potential evasion mechanisms of cancer cells as a consequence of drug treatment. The sunitinib example illustrates that drawing conclusions from non-predictive animal models—apart from wasting research funding and animal lives—might expose patients to unnecessary risk.

The second category of discrepancy reasons discussed by Sena et al. is a more general issue. Animal studies often seem to lack certain aspects of study design which are fully established in clinical trials, e.g. randomization of test subjects to treatment or control groups, blinded performance of treatment and blinded assessment of outcome. Such study design aspects seem to lead to overestimated drug efficacy in preclinical animal research if neglected [4, 9, 12–14].

## Underpowered studies

For a scientific efficacy confirmation of drug candidates or other medical interventions, statistical testing is performed to identify significant differences between test groups. For publication of biomedical research data, most journal editors and peer reviewers request statistical significance of results. The sense and suitability of this practice has often been criticized [15, 16], but this topic will not be elaborated here in further detail. In fact, statistical significance is dependent mainly on the underlying null hypothesis, the sample size, the variance of the data and the significance level alpha, which in most cases is taken as the 5 % limit somewhat arbitrarily introduced by Ronald Fisher. Generally speaking, the bigger the group size, the easier it gets to find statistical differences between two test groups. Or similarly, the more homogeneous the test groups, the higher the chance to find statistical differences between the groups for reasons of decreased data variance.

In order to reliably find significant differences between test groups, it is necessary to carefully determine the size of a relevant effect and estimate the expected variance of the data. Based on these two parameters (minimal effect size and variance) it is possible to calculate the minimal size of test groups required to detect statistical differences with a certain probability. This probability is referred to as the power or sensitivity of the study and by convention should be at least 80–90 %. Systematic reviewing of preclinical stroke data has shown that such considerations together with sample size calculation in the planning phase of a study are hardly ever performed [9, 12]. In preclinical animal research in general, ethical concerns, deservedly so, play an important role. Group sizes in animal experimentation are therefore requested by authorities to be kept as small as possible. As a consequence, many studies report small animal group sizes. Such studies are often underpowered, as it is not possible to reliably detect group differences with high enough probability. Results of underpowered studies are in fact useless and therefore just as unethical as using more animals per experiment than necessary.

For medical progress it is essential that experiments yield reliable data. Results need to be externally valid for successful extrapolation from the bench to the bedside. Underpowered studies in this regard suffer from a major problem: the chance to detect valid effects is too low to be reliable in any kind. In an attempt to outweigh power problems due to small group sizes, test parameters, including test animals, are usually standardized as much as possible to reduce data variance, e.g. test animals are often inbred. It is widely assumed that such strict standardizations increase the reproducibility and therefore the reliability of research data [17].

## Standardization fallacy

Several years ago, Crabbe et al. tried to test reproducibility of experimental animal data by comparing behavioural tests performed in different laboratories in North America [18]. The researchers tried to strictly standardize all possible parameters of their experiments they could think of between these laboratories. Animals were for example shipped on the same day from the same supplier or experimenters wore gloves of the same kind in all three laboratories. Mice of different strains underwent several behavioural tests, e.g. either after cocaine or vehicle injection. Crabbe and his colleagues were assuming that in such a maximally standardized setup reproducibility of results should be very high. Surprisingly, this was not the case, as they found large effects of site in mouse behaviour. Especially small genetic effects turned out to be strongly influenced by environmental factors and animal handling. Wahlsten and colleagues concluded in a follow-up article that it might be impossible to adjust the laboratory environment so that it is sufficiently similar between facilities to guarantee highest possible reproducibility of results [19]. In another study including multiple laboratories, Richter and colleagues tried to investigate whether strict standardization of experimental parameters beneficially affects reproducibility of mouse behaviour [17]. They retrospectively regrouped test animals into “pseudo-heterogenized” groups and compared the observed behavioural strain differences with the original strictly standardized samples in terms of false-positive result detection, i.e. non-reproducible results with no external validity. Richter and coworkers concluded that overly strict standardization of environmental parameters might lead to spurious results with no external validity. This had earlier been referred to as the “standardization fallacy” [20]. The authors therefore suggest that adequate environmental heterogenization of such factors might improve reproducibility of results. They later confirmed their results empirically [21].

## Translational research in nuclear medicine

The question arises now to what extent such problems also apply to the field of nuclear medicine and molecular imaging. It can be argued that mouse behaviour might be less robustly measured than the distribution of a PET tracer in the mouse body. Furthermore, it is well conceivable that mouse behaviour, as an enormously complex outcome measure, might be more prone to environmental influences than the uptake of a specific PET tracer into tumour tissue of mice. In an attempt to extrapolate the study design of Richter and colleagues to the field of small animal PET, we recently performed a pilot study to investigate the impact of strict standardization on the generation of false-positive results [22]. We assessed ^18^F-fluorodeoxyglucose (FDG) tissue uptake differences between male and female NMRI mice. A statistically significant confirmation of the Richter study was not possible, presumably due to small study power, but we found a clear trend towards higher false-positive rates under strictly standardized experimental conditions compared to pseudo-heterogenized ones. Furthermore, we tried to assess the inherent variability of ^18^F-FDG brain uptake in SD rats under highly standardized conditions. We determined uptake variability within animals using a test-retest approach, as well as uptake variability between different individuals of the same test group (intra- vs inter-animal variability). Contrary to our assumptions that intra-animal variability would be essentially smaller than inter-individual variability, we found that both types of variability were in the same range. Despite the small scale of our study and hence the resultant shortcomings, the data affirm that reproducibility of animal experiments is a problem in nuclear medicine as well as in behavioural mouse research and we are convinced that further research in this regard seems worthwhile. In addition, we believe that the development of new interventions in nuclear therapy or diagnostics might suffer from translation problems similar to drug development in acute stroke, which was reported by Sena and colleagues [9]. Even though to our knowledge never formally investigated, it seems conceivable that a substantial fraction of preclinical animal data in the field of molecular imaging might be misleading. Exemplarily, the development of a new PET tracer for pancreatic tumour diagnosis shall be considered. In the majority of in vivo proof of principle research, mice with subcutaneously inoculated pancreas tumour xenografts are investigated. Such tumours grow, isolated from their usually occurring stromal surrounding, at a well-exposed body part, e.g. the shoulder. It seems logical that a PET scan visualizes even rather small effects of tumour uptake, as the tumour surrounding presumably does not accumulate enough radiotracer to diminish the contrast of tumour uptake. Such an artificial model situation, in our understanding, cannot represent or reflect, let alone predict the tumour visualization capacity of the new compound in patients reliably, as the tissues surrounding spontaneously occurring pancreatic tumours in the abdominal region often show high accumulation of radiotracers, e.g. due to excretion processes.

We assume that translation of preclinical animal data to the clinic, apart from the inevitable species differences, often fails due to avoidable quality problems, which partially depend on the field of research (e.g. disease models or lack of comorbidities). If such shortcomings could be systematically assessed, we are convinced that the reliability of preclinical in vivo data could be increased crucially. This includes better design of animal models as well as higher general study quality, such as blinding, randomization or avoiding underpowered studies. Such improvements would meet highest ethical standards, even if individual experimental group sizes might be bigger.

## Publication bias

In our opinion, a systematic status quo assessment of preclinical animal research seems adequately achievable by systematic reviewing and meta-analysis. Nevertheless, systematic reviewing and meta-analysis depend on the complete availability of all research performed regarding a particular topic. As long as results are incompletely published, systematic reviews and meta-analyses are liable to bias. This bias is referred to as publication bias, i.e. favouring publication of positive or confirming data over negative or inconclusive data [23]. This overweighting of positive results leads to a subsequent overestimation of effect sizes in meta-analyses and systematic reviews. The publication bias problem has been acknowledged in clinical research [23–25]. In animal research, publication bias has been less thoroughly investigated but there is evidence that researchers are becoming increasingly aware of it. In a recent publication, Sena and colleagues came to the conclusion that publication bias accounts for one third of the effect in animal stroke studies that was assessed in a systematic review [26]. A Dutch research team recently tried to assess the opinion amongst Dutch animal researchers and find possible reasons for the publication bias phenomenon (ter Riet et al., personal communication). Interestingly, journal editors and peer reviewers were regarded as prominent drivers for non-publication of negative or non-significant research data. The authors suggest strategies to address publication bias, such as mandatory publication of study protocols, research results or the reasons why no results were obtained. According to ter Riet and colleague’s study, Dutch researchers think that these strategies might help to increase scientific progress.

### Conclusion

We believe that preclinical animal research needs to improve on different levels to yield best predictions for human patients. Animal models of diseases need to be as reliably reflective of the patient’s situation as possible and it should be considered to what extent experimental parameters need to be homogenized or if a controlled heterogenization might be appropriate. Furthermore, general aspects of experimental planning and performance should be brought to a higher level. Regulatory authorities as well as journal editors and peer reviewers should pay increasing attention to features like sample size calculation, blinding and randomization. And finally, efforts should be taken to reduce publication bias in animal research literature to increase the reliability of meta-analytic analysis of studies in order to identify reasons for insufficient prediction of models. Only if joint efforts are made is there hope that preclinical animal research will yield safe and maximally predictive results for the clinic. This concerns us all: researchers in phases of experimental planning and performance as well as regulatory authorities, journal editors and peer reviewers.
